# HBV activates hepatic stellate cells through RUNX2/ITGBL1 axis

**DOI:** 10.1186/s12985-025-02749-z

**Published:** 2025-04-26

**Authors:** Fengchun Shi, Wei Tan, Wei Huang, Fei Ye, Mingjie Wang, Yongxiang Wang, Xinxin Zhang, Demin Yu

**Affiliations:** 1 Department of Infectious Diseases, Research Laboratory of Clinical Virology, Ruijin Hospital, Shanghai Jiao Tong University School of Medicine, Shanghai, 200025 China; 2 Department of Gastroenterology, Affiliated Hospital of Nantong University, Nantong, 226000 China; 3 Department of Infectious Diseases, Xiangya Hospital Central South University, Changsha, 410008 Hunan Province China; 4 Department of Gastroenterology, Ruijin Hospital, Shanghai Jiao Tong University School of Medicine, Shanghai, 201821 China; 5 Key Laboratory of Medical Molecular Virology (MOE/NHC/CAMS), Shanghai Frontier Science Center of Pathogenic Microorganisms and Infection, School of Basic Medical Sciences, Shanghai Medical College, Fudan University, Shanghai, China

**Keywords:** Chronic hepatitis B, RUNX2, ITGBL1, Liver fibrosis

## Abstract

**Background:**

Chronic hepatitis B (CHB) remains a global health challenge, with liver fibrosis serving as a critical determinant of disease progression. Despite antiviral treatments, liver fibrosis often persists in CHB patients, highlighting the need for additional biomarkers and therapeutic targets. This study investigates the molecular mechanism underlying HBV-induced liver fibrosis, focusing on the role of RUNX2 in regulating integrin beta-like 1 (ITGBL1), a key factor in fibrogenesis.

**Methods:**

We examined the relationship between RUNX2 and ITGBL1 in both in vitro hepatocyte models and an in vivo HBV mouse model. Using chromatin immunoprecipitation (ChIP), luciferase reporter assays, and Western blotting, we assessed RUNX2 binding to the ITGBL1 promoter and its impact on gene expression. We also evaluated the effects of RUNX2 inhibition using Vitamin D3 and CADD522 on ITGBL1 expression and hepatic stellate cell activation.

**Results:**

Our findings reveal that RUNX2 directly binds to the ITGBL1 promoter, enhancing its expression and promoting hepatic stellate cell activation. We show that HBV infection significantly upregulates both RUNX2 and ITGBL1 in liver cells. Inhibition of RUNX2 with Vitamin D3 or CADD522 significantly reduced ITGBL1 levels and blocked hepatic stellate cell activation. These results suggest that the RUNX2/ITGBL1 pathway is critical in the progression of liver fibrosis in HBV-infected patients.

**Conclusions:**

RUNX2 promotes liver fibrosis in HBV-infected patients by upregulating ITGBL1 expression. Our findings suggest that targeting RUNX2 could be a potential therapeutic approach to mitigate liver fibrosis in chronic hepatitis B.

**Supplementary Information:**

The online version contains supplementary material available at 10.1186/s12985-025-02749-z.

## Introduction

Even with antiviral therapy and vaccination, HBV infection remains a global health problem. Approximately 296 million individuals are affected by HBV worldwide, and around one million deaths occur annually due to HBV-related diseases such as hepatic decompensation and hepatocellular carcinoma (HCC) [1]. Liver fibrosis is an important factor impacting the prognosis of chronic hepatitis B (CHB) patients. While CHB patients achieving sustained virological response through antiviral therapy can experience fibrosis reversal, several patients who do not attain sustained virological response remain at high risk for liver fibrosis [2–4]. However, the mechanisms underlying HBV-related liver fibrosis are not yet fully understood.

Integrin beta-like 1 (ITGBL1) encodes a protein containing ten integrin epidermal growth factor (EGF)-like repeat domains. Previous studies have demonstrated that ITGBL1 is associated with tumor invasion and metastasis. Recent research suggests that ITGBL1 plays a pivotal role in the development and progression of fibrosis across various tissues, including the heart, lung, and liver [5–9]. Our previous studies analyzed liver biopsy expression profiles from 128 CHB patients and identified ITGBL1 as the gene most strongly associated with fibrosis stage. We demonstrated that ITGBL1 stimulates hepatic stellate cells (HSCs) to promote liver fibrosis by activating the TGF-β pathway [9], and we also found that serum ITGBL1 levels in CHB patients were significantly higher than those in healthy controls [10]. However, the mechanism by which HBV affects ITGBL1 expression remains unclear.

Runt-related transcription factor 2 (RUNX2), a DNA-binding transcription factor located at 6p21.1 in humans, orchestrates the differentiation of osteoblasts and chondrocytes by regulating multiple signaling pathways [11]. RUNX2 has been reported to be associated with multi-organ fibrosis; for example, in aortic valve interstitial cells, RUNX2 activation leads to increased production of fibrotic proteins MMP-9 and collagen I [12]. In liver fibrosis, RUNX2 has been reported to activate hepatic stellate cells (HSCs) by upregulating Itgav expression and functioning as a transcriptional inhibitor of SLC27A5, resulting in the buildup of cholic acid and subsequent activation of HSCs. Increased RUNX2 expression is confirmed in many liver diseases such as NAFLD and alcoholic hepatitis [13, 14]. Studies have shown that RUNX2 regulates ITGBL1 in breast cancer, colon cancer, and melanoma [15–17]. Based on these observations, we hypothesize that HBV may promote fibrosis progression by upregulating RUNX2, which in turn modulates ITGBL1 expression.

In this study, we examined the regulatory relationship between HBV, RUNX2, and ITGBL1 in liver cells and an rAAV-HBV mouse model. Our results collectively establish that HBV promotes fibrosis through the RUNX2-mediated upregulation of ITGBL1, highlighting potential therapeutic targets for CHB-related liver fibrosis.

## Materials and methods

### Plasmids

The expression plasmids carrying the ITGBL1 open reading frame (ORF) and RUNX2 ORF were obtained from Genecopoeia (GeneCopoeia, Rockville, MD, USA). The ITGBL1 promoter luciferase reporter plasmid was also purchased from Genecopoeia. The small hairpin RNA plasmids interfering with the expression of RUNX2 were purchased from Genecopoeia. The target sequences of shRNA are shown in Supplementary Table S1. The wild-type plasmid (pWT) contains a 1.2× copy of the genotype C HBV genome with terminal redundancy [18]. The HBx expression plasmid (pCDH-HBx) and the HBs expression plasmid (pCDH-HBs) were generously provided by Professor Yong Xiang Wang (Fudan University, Shanghai, China).

### Cell culture, animals, transduction, and transfection

HEK293T, Huh7, LX-2, and AD38 cells were cultured in Dulbecco’s Modified Eagle Medium (DMEM) supplemented with 10% fetal bovine serum, 100 mg/mL penicillin, 10 ng/mL streptomycin, and 2 mmol/L L-glutamine (GIBCO-BRL, Co. Ltd., USA) at 37 °C in 5% CO₂. Transfection was performed using X-tremeGENE 9 DNA Transfection Reagent (Roche) according to the manufacturer’s instructions. Briefly, cells were seeded in six-well plates at a density of 5.0 × 10⁵ cells/well 12 h before transfection. The DNA to transfection reagent ratio was 1:3. X-tremeGENE 9 was diluted in 100 µL Opti-MEM Reduced Serum Medium (GIBCO-BRL, Co. Ltd., USA), and DNA was added. The mixture was incubated at room temperature for 15 min before being added to the cells. Cells were incubated with the transfection mixture at 37 °C in 5% CO₂.

To establish the AAV-HBV mouse model, 6-week-old male C57BL/6 mice were randomly divided into three groups (*n* = 3 per group): HBV-infected group, mock-infected control (empty vector), and untreated control group. Mice in the HBV group received tail vein injections containing 5 × 10¹⁰ viral genome equivalents (vge) of rAAV8–1.3×HBV diluted in 200 µL saline. The mock group received 5 × 10¹⁰ vge of empty AAV8 diluted in 200 µL saline. The untreated control group received no intervention. Blood samples (200 µL) were collected at weeks 0, 1, 4, and 8 post-injection via retro-orbital bleeding for serum ITGBL1 and HBV DNA quantification. At week 8, all mice were euthanized, and liver tissues were harvested for immunohistochemical analysis of ITGBL1 expression.

All animal experiments were conducted in accordance with local animal welfare regulations and the guidelines for the care and use of laboratory animals. The study received approval from the Animal Ethics Committee of the School of Basic Medical Sciences, Fudan University (Approval No. 20210302-103).

### RNA extraction and real-time PCR

Total RNA extraction was performed using TRNzol Universal Reagent (Tiangen Biotech (Beijing) Co., Ltd.) according to the manufacturer’s instructions. Reverse transcription and RT-PCR were performed using the Hifair^®^ II 1st Strand cDNA Synthesis SuperMix for qPCR (gDNA digester plus) Kit and Hieff UNICON^®^ qPCR SYBR Green Master Mix (Yeason Biotech (Shanghai) Co., Ltd.), respectively. The amplification parameters were 95 °C for 30 s, followed by 40 cycles of 95 °C for 10 s and 60 °C for 30 s. GAPDH was used as the internal control. The relative expression of α-SMA and COL1A1 mRNA was calculated using the ΔΔCt (2 − ΔΔCt) method. The primer sequences for α-SMA and COL1A1 are listed in Supplementary Table S2.

### HBV DNA and viral antigen quantification

HBV DNA levels in mouse serum and in the supernatant of AD38 cell culture were quantified using a real-time PCR assay (Shanghai Kehua Bio-Engineering Co., Ltd.) with a lower limit of detection of 500 IU/mL. Quantitative HBsAg levels in the culture medium of Huh7 cells and the presence of HBeAg were measured using the Abbott Architect immunoassay system (Abbott Laboratories, IL, USA), according to the manufacturer’s instructions.

### Western blot analysis

Proteins were extracted using RIPA lysis buffer (Thermo Fisher) containing protease inhibitor cocktail (Thermo Fisher). Total proteins were separated by 12.5% SDS-polyacrylamide gel electrophoresis, and the proteins were transferred to NC membranes using an eBlot L1 system (Genscript). The membranes were blocked using QuickBlock™ Blocking Buffer (Beyotime) and incubated with primary antibodies: anti-ITGBL1 (1:1,000, Abcam, cat. no. ab93592), anti-RUNX2 (1:1,000, Abcam, cat. no. ab53707), and anti-Lamin-B1 Rabbit mAb (1:1,000, Cell Signaling Technology, cat. no. AC057). Secondary antibodies were HRP-labeled Goat Anti-Rabbit IgG(H + L) (Beyotime). The immunoblot signals were visualized and quantified using the Tanon-5200 Chemiluminescent Imaging System (Tanon).

### ELISA for serum ITGBL1 measurement

ELISA was performed using a commercial ITGBL1 ELISA Kit (Jiangsu Jingmei Biotechnology) according to the manufacturer’s instructions. Briefly, 50 µL of standard solutions and serum samples were added to the pre-coated wells. After incubation and washing, the HRP-conjugated secondary antibody was added, followed by chromogenic substrate development. Absorbance at 450 nm was measured using a microplate reader (RT-6100, Rayto).

### Chromatin Immunoprecipitation (ChIP) assay

Huh7 cells were cross-linked with 1% paraformaldehyde for 10 min at room temperature. The cells were lysed and sonicated at 30% power for 10 cycles (15 s ON, 15 s OFF). After sonication, the supernatants were incubated with anti-RUNX2 (Abcam, cat. no. ab236639) antibody or control Rabbit IgG (Beyotime, A7016) overnight at 4 °C. Chromatin-antibody complexes were captured using protein A/G magnetic beads (Millipore), washed, and then eluted. Real-time PCR was performed to analyze RUNX2-binding DNA fragments from the ChIP assay. Primers for qPCR are listed in Table S2 (Supporting Information).

### Luciferase reporter assay

HEK293T cells were transfected in 96-well plates with the appropriate plasmids and treated with inhibitors as necessary. Cells were harvested 48 h post-transfection, and luciferase activity was assayed using the Dual-Luciferase Assay Kit (GeneCopoeia). All experiments were performed at least three times, and data are expressed as mean ± SEM.

### Liver tissue immunohistochemistry

The liver tissues of mice were fixed in 10% formalin and subjected to immunohistochemistry (IHC) analysis. The antibody used for IHC was anti-ITGBL1 (Abcam, cat. no. ab93592).

### Data collection and preprocessing

The GEO database (http://www.ncbi.nlm.nih.gov/geo) was used to obtain the gene expression profiles. The gene expression profile of GSE84088 was downloaded [9]. Pre-processing programs (including background adjustment, normalization, summarization, and gene probe annotation) were executed using R language (Version 4.4.2; https://www.R-project.org).

### Statistical analysis

Statistical analyses were performed using R version 4.4.2 (https://www.R-project.org). Data with a normal distribution are presented as the mean ± standard deviation (SD) and compared using Student’s *t*-test. Pearson correlation coefficients were calculated to assess the relationships between ITGBL1, RUNX2, and HBV DNA levels in cross-sectional data.

Repeated measures were analyzed using one-way or two-way analysis of variance (ANOVA), followed by Tukey’s post hoc test where appropriate.

In addition, **repeated measures correlation analysis** was conducted using the rmcorr package in R to evaluate within-subject correlations between HBV DNA levels and ITGBL1 or RUNX2 expression across multiple time points.

To account for inter-individual variability in repeated measurements, **linear mixed-effects models (LMMs)** were applied using the lme4 and lmerTest packages, with Mouse ID included as a random intercept. Statistical significance was defined as *p* < 0.05.

## Result

### HBV enhances ITGBL1 expression in hepatoma cells and mouse models

**Fig. 1:**
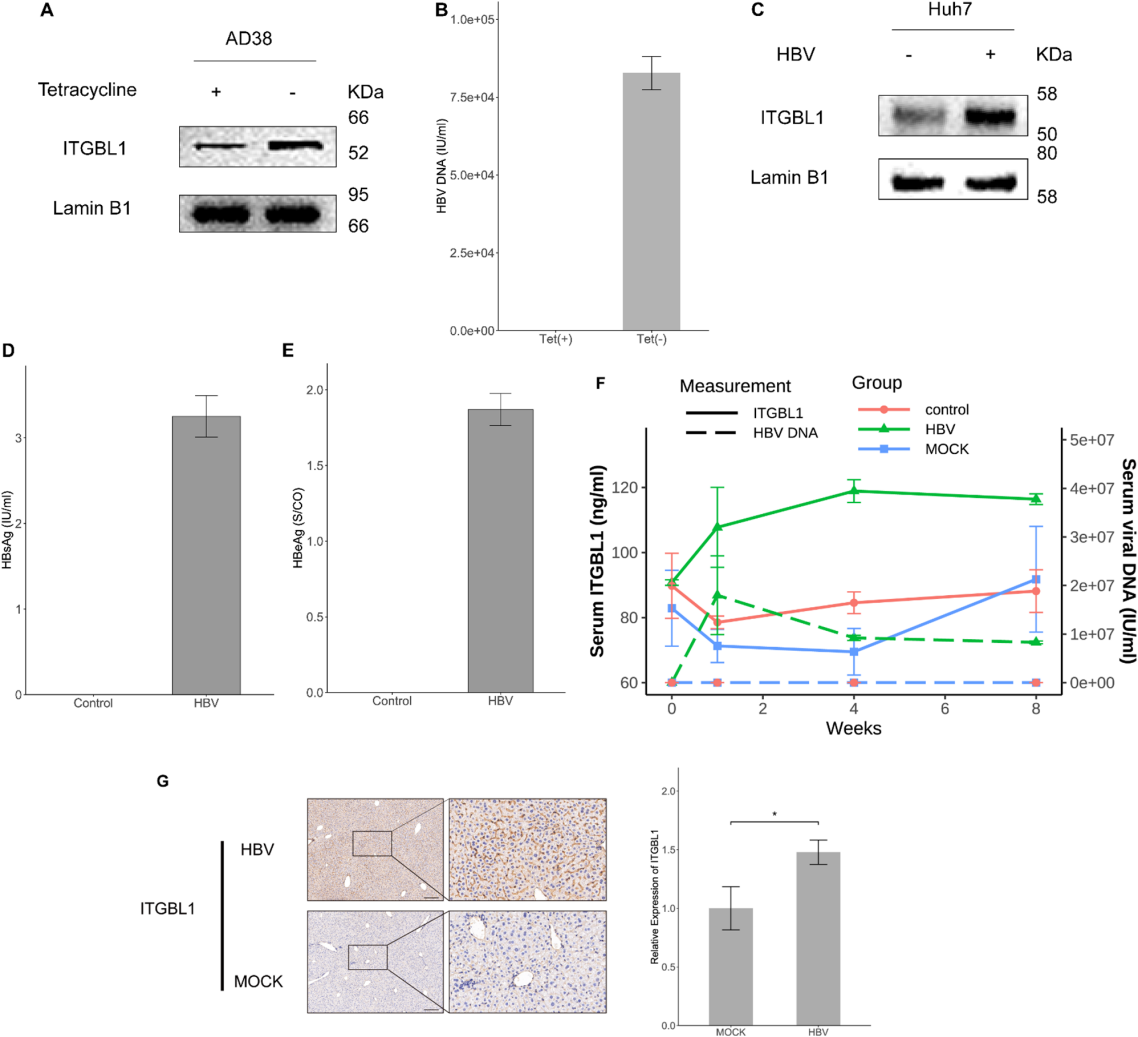
Expression of ITGBL1 is increased in the HBV expression model. (**A**) ITGBL1 protein levels were elevated following the induction of HBV replication in HepAD38 cells. Cells were cultured in the presence (1 µg/mL) or absence of tetracycline for 48 h. Western blot analysis revealed increased ITGBL1 expression under HBV-expressing conditions (tetracycline withdrawal). (**B**) HBV DNA levels in the supernatant of HepAD38 cells were quantified by real-time PCR. HBV DNA levels were significantly elevated in the tetracycline-free group compared to the tetracycline-treated group, indicating effective induction of HBV replication. Data are presented as mean ± SEM from three independent experiments. (**C**) Huh7 cells were seeded in 6-well plates and transfected with 1 µg of pHBV1.2 plasmid. Cells were harvested 48 h post-transfection, and ITGBL1 expression was analyzed by Western blot. A significant increase in ITGBL1 protein levels was observed in HBV-transfected Huh7 cells compared to control cells. (**D**–**E**) HBsAg and HBeAg levels in the supernatant of HBV-transfected Huh7 cells were quantified using the Abbott Architect immunoassay system. Since residual plasmid DNA in the culture medium after transfection could interfere with HBV DNA quantification—even after DNase treatment—viral protein expression was used as an indicator of successful HBV transfection. To investigate the effect of HBV on intrahepatic ITGBL1 expression, two groups of 6-week-old male C57BL/6 mice were intravenously injected with 200 µL saline containing either 5 × 10¹⁰ viral genome equivalents (vge) of rAAV8–1.3×HBV or empty rAAV8, respectively, as described in the Materials and Methods section. A control group of untreated mice was also included. Blood samples (200 µL) were collected at weeks 0, 1, 4, and 8 post-injection via retro-orbital bleeding for the analysis of serum ITGBL1 and HBV DNA levels. Each group consisted of three mice (*n* = 3). (**F**) Serum ITGBL1 levels were measured using an ELISA kit, and HBV DNA levels were quantified using a real-time PCR assay (Shanghai Kehua Bio-Engineering Co., Ltd.) with a lower limit of detection of 500 IU/mL. (**G**) At week 8 post-injection, mice in the MOCK and HBV groups were sacrificed, and liver tissues were collected for immunohistochemistry (IHC) using an anti-ITGBL1 antibody. Representative images demonstrate increased ITGBL1 expression in the liver sections of rAAV8–1.3×HBV-infected mice compared to controls. Relative staining intensity was quantified using ImageJ software. Data are presented as mean ± SEM; *p* < 0.05 vs. control group. Scale bar: 200 μm (*n* = 3)

To investigate the impact of HBV on ITGBL1 expression, we initially utilized an inducible HBV expression system in HepAD38 cells. Western blot analysis revealed that ITGBL1 protein levels were significantly elevated upon removal of tetracycline, indicating upregulation of ITGBL1 under HBV-replication conditions (Fig. 1A). Concurrently, real-time PCR analysis demonstrated increased HBV DNA levels in the culture supernatant following tetracycline withdrawal, confirming effective induction of HBV replication (Fig. 1B).

To further validate this finding, Huh7 cells were transiently transfected with the HBV-replicative plasmid. As shown in Fig. 1C, ITGBL1 expression was markedly upregulated in HBV-transfected Huh7 cells compared with control cells. Levels of HBsAg and HBeAg in the supernatant were measured using the Abbott Architect immunoassay system, serving as indicators of successful HBV replication, given that residual plasmid DNA in the culture supernatant may interfere with qPCR result of HBV DNA — even after DNase treatment (Fig. 1D–E).

To evaluate the in vivo impact of HBV on ITGBL1 expression, serum levels of ITGBL1 and HBV DNA were measured at multiple time points following rAAV8–1.3×HBV administration. A progressive increase in both serum ITGBL1 and HBV DNA levels was observed in the rAAV8–1.3×HBV group compared to controls, as determined by ELISA and quantitative real-time PCR (Fig. 1F).

Consistently, immunohistochemical analysis of liver tissues at week 8 revealed markedly elevated ITGBL1 expression in the livers of HBV-infected mice relative to the Mock group. Quantification of staining intensity confirmed a significant increase in intrahepatic ITGBL1 expression (Fig. 1G).

These findings collectively demonstrate that HBV infection enhances ITGBL1 expression in vivo, supporting the results observed in hepatoma cell models.

### RUNX2 regulates ITGBL1 expression in liver cells

**Fig. 2:**
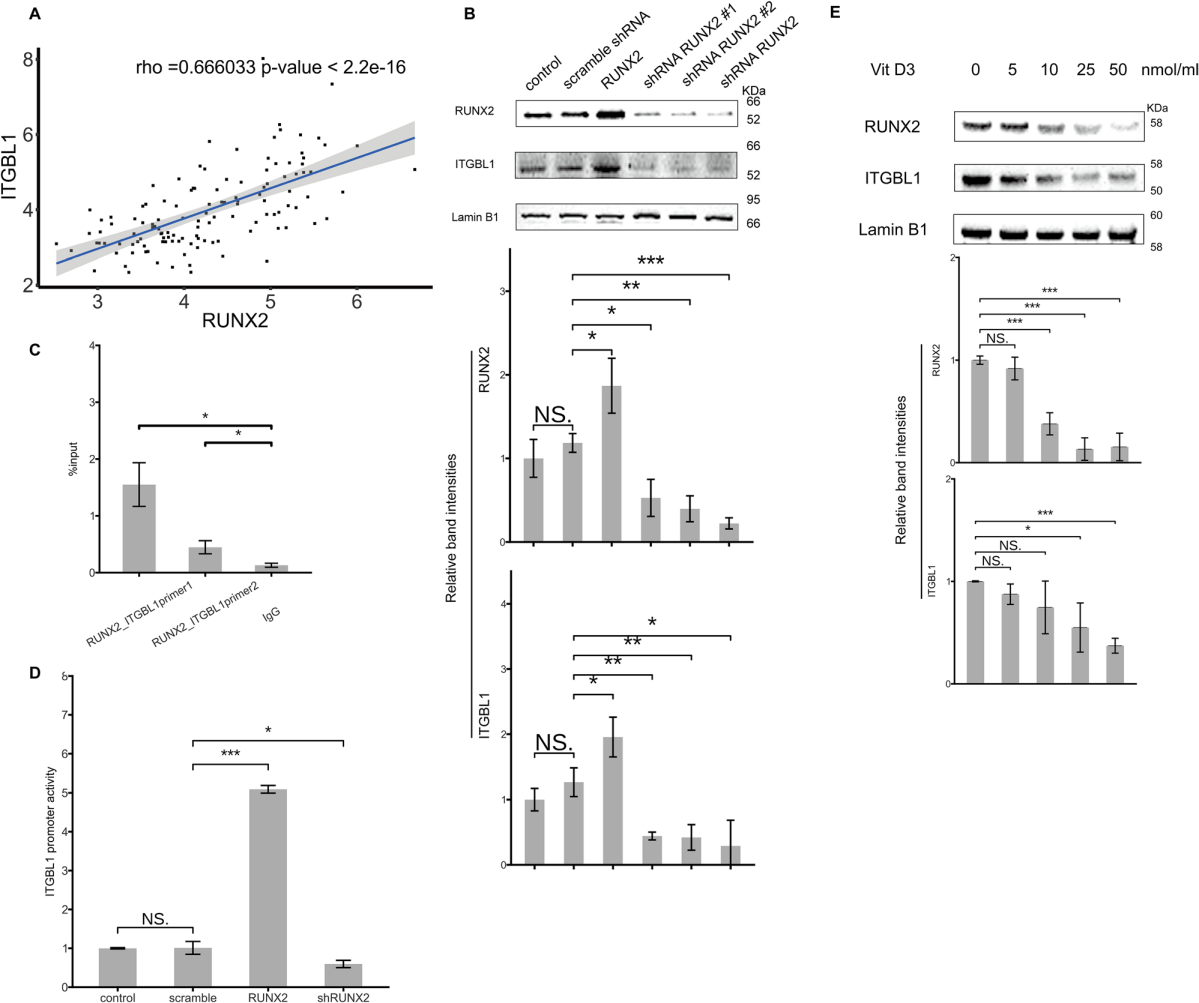
The expression of ITGBL1 in hepatocytes is regulated by RUNX2. (**A**) The mRNA expression data from liver biopsies of CHB patients in the GSE84044 cohort were analyzed using Pearson correlation coefficients to assess the relationship between ITGBL1 and RUNX2. A significant positive correlation was observed between ITGBL1 and RUNX2 (Pearson’s *r* = 0.66603, *p* < 0.01). (**B**) Huh7 cells seeded in 6-well plates were transfected with RUNX2 overexpression plasmid, shRUNX2 plasmid, and shscramble plasmid as a negative control, with untreated cells as control. Cells were harvested 48 h post-transfection, and Western Blot was used to analyze the expression of ITGBL1 and RUNX2. In Huh7 cells, the expression of ITGBL1 was enhanced with RUNX2 overexpression and downregulated with RUNX2 knockdown. (**C**) ChIP experiments in Huh7 cells showed that RUNX2 binds to the ITGBL1 promoter. (**D**) The ITGBL1 promoter plasmid and shRUNX2 plasmid were co-transfected into HEK293T cells (96-well plate), and the transcriptional activity of the ITGBL1 promoter was detected by dual-luciferase reporter assays 48 h after co-transfection. The activity of the ITGBL1 promoter increased with RUNX2 overexpression and decreased with RUNX2 knockdown. (**E**) Huh7 cells seeded in 6-well plates were treated with increasing concentrations of the RUNX2 inhibitor, Vitamin D3. After 48 h of treatment with Vitamin D3, Western Blot was used to detect the expression of ITGBL1 and RUNX2. As the concentration of Vitamin D3 increased, RUNX2 expression gradually decreased, and the expression of ITGBL1 was downregulated in accordance with the reduction in RUNX2 expression. The figure shows a representative image

Previous studies have reported that RUNX2 induces ITGBL1 expression in breast cancer and melanoma. To determine whether RUNX2 similarly regulates ITGBL1 in liver tissues, we performed gene correlation analysis using the expression profiling array of liver biopsy samples from CHB patients (dataset GSE84044). Clinical characteristics of patients, i.e., HBV DNA levels, fibrosis staging scores, and HBeAg/HBsAg serostatus, are available in the supplementary materials of the study article [9]. The analysis revealed a significant correlation between the expression of ITGBL1 and RUNX2 (Fig. 2A). Further, to validate the regulatory relationship between RUNX2 and ITGBL1, we knocked down RUNX2 using shRNA and overexpressed RUNX2 in Huh7 cells. Knockdown of RUNX2 resulted in a significant decrease in basal ITGBL1 expression, whereas overexpression of RUNX2 led to a notable increase in ITGBL1 protein levels (Fig. 2B). Given that RUNX2 is a DNA-binding transcription factor, chromatin immunoprecipitation (ChIP) assays in Huh7 cells confirmed RUNX2 binding to the ITGBL1 promoter (Fig. 2C). Furthermore, dual-luciferase reporter assays demonstrated that overexpression of RUNX2 significantly enhanced ITGBL1 promoter activity, while RUNX2 knockdown significantly reduced promoter activity (Fig. 2D). To further confirm RUNX2’s regulation of ITGBL1, we treated Huh7 cells with increasing concentrations of Vitamin D3, an inhibitor of RUNX2 [19]. As shown in Fig. 2E, along with increasing doses of Vitamin D3, RUNX2 expression was downregulated accordingly, accompanied by a reduction in ITGBL1 levels. These findings suggest that RUNX2 upregulates ITGBL1 protein levels by binding to the ITGBL1 promoter and enhancing its activity in liver cells.

### HBV upregulates ITGBL1 expression dependent on RUNX2

**Fig. 3:**
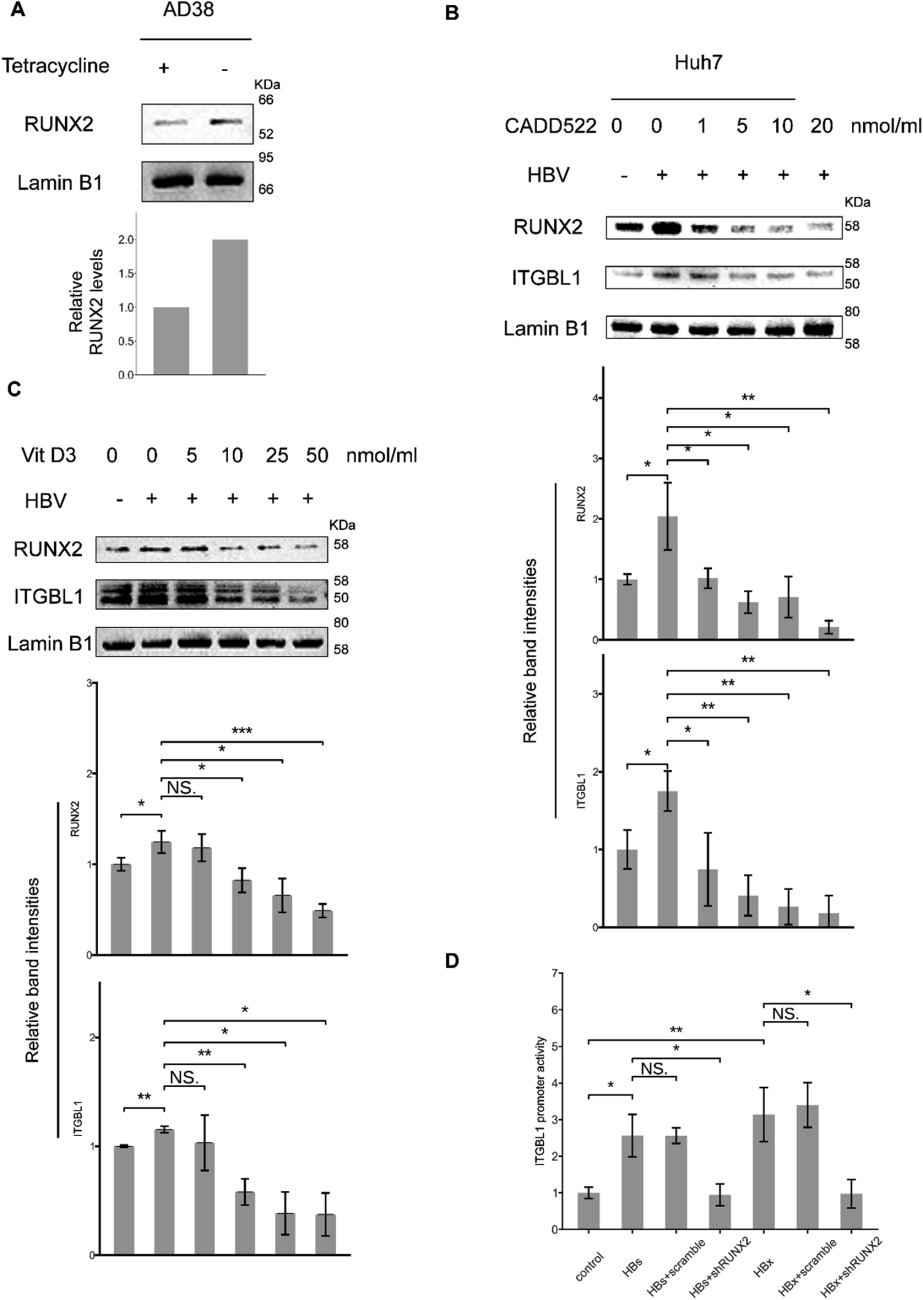
HBV promotes ITGBL1 expression by upregulating RUNX2. (**A**) RUNX2 protein level elevation after the induction of HBV expression in HepAD38 cells. (**B**) Huh7 cells seeded in 6-well plates were transfected with 1 µg of pHBV1.2 and treated with increasing concentrations of the RUNX2 inhibitor CADD522. Cells were harvested 48 h post-transfection. RUNX2 expression was elevated in HBV-expressing Huh7 cells, and as the concentration of CADD522 increased, RUNX2 expression was downregulated. The upregulation of ITGBL1 by HBV was also blocked by the RUNX2 inhibitor. (**C**) Huh7 cells seeded in 6-well plates were transfected with 1 µg of pHBV1.2 and treated with increasing concentrations of the RUNX2 inhibitor Vitamin D3. Cells were harvested 48 h post-transfection. RUNX2 expression was elevated in HBV-expressing Huh7 cells, and as the concentration of VitD3 increased, RUNX2 expression was downregulated. The upregulation of ITGBL1 by HBV was also blocked by the RUNX2 inhibitor. (**D**) Since no difference in ITGBL1 expression was observed between patients with positive and negative hepatitis B e antigen (HBeAg), the ITGBL1 promoter plasmid and HBs and HBx expression plasmids were co-transfected into HEK293T cells (96-well plate), respectively, and the transcriptional activity of the ITGBL1 promoter was detected by dual-luciferase reporter assays 48 h after co-transfection. Expression of HBx and HBsAg promoted the activity of the ITGBL1 promoter, while interference with RUNX2 blocked the upregulation of ITGBL1 promoter activity. The figure shows a representative image

We observed that, in HepAD38 cells, RUNX2 expression was significantly elevated in the absence of tetracycline, compared to in the presence of tetracycline, which prompted us to further investigate the mechanistic relationship between RUNX2 and ITGBL1 in the context of HBV infection (Fig. 3A). We inhibited RUNX2 expression in Huh7 cells transfected with the pWT plasmid using increasing doses of CADD522, a specific RUNX2 inhibitor. The results showed a corresponding dose-dependent decrease in ITGBL1 levels (Fig. 3B). This reduction in ITGBL1 upon RUNX2 inhibition indicates that RUNX2 is a critical regulator of ITGBL1 expression in HBV-related liver cells.

Furthermore, to confirm that RUNX2 is essential for HBV upregulation of ITGBL1 expression, we treated HBV-transfected Huh7 cells with progressively increasing doses of Vitamin D3, known to inhibit RUNX2 activity. Similar to the CADD522 treatment, Vitamin D3 administration led to a significant decrease in ITGBL1 expression in a dose-dependent manner (Fig. 3C). These consistent results from two independent RUNX2 inhibition methods strengthen the evidence that HBV induces ITGBL1 expression in a RUNX2-dependent manner.

Furthermore, to elucidate the direct interaction between RUNX2 and the ITGBL1 promoter in the context of HBV, we conducted a dual-luciferase reporter assay. The results demonstrated that HBV enhances the luciferase activity of the ITGBL1 promoter, indicating transcriptional activation. However, when RUNX2 was attenuated by shRNA, HBV failed to promote ITGBL1 promoter activity, suggesting that RUNX2 is necessary for HBV-mediated transcriptional activation of ITGBL1 (Fig. 3D). Collectively, these findings indicate that RUNX2 is necessary for HBV-induced upregulation of ITGBL1, as RUNX2 directly binds to the ITGBL1 promoter to enhance its activity in liver cells. This RUNX2-dependent regulation of ITGBL1 underscores the potential of targeting RUNX2 as a therapeutic strategy in HBV-related liver pathogenesis.

### HBV promotes activation of hepatic stellate cells via RUNX2/ITGBL1

**Fig. 4:**
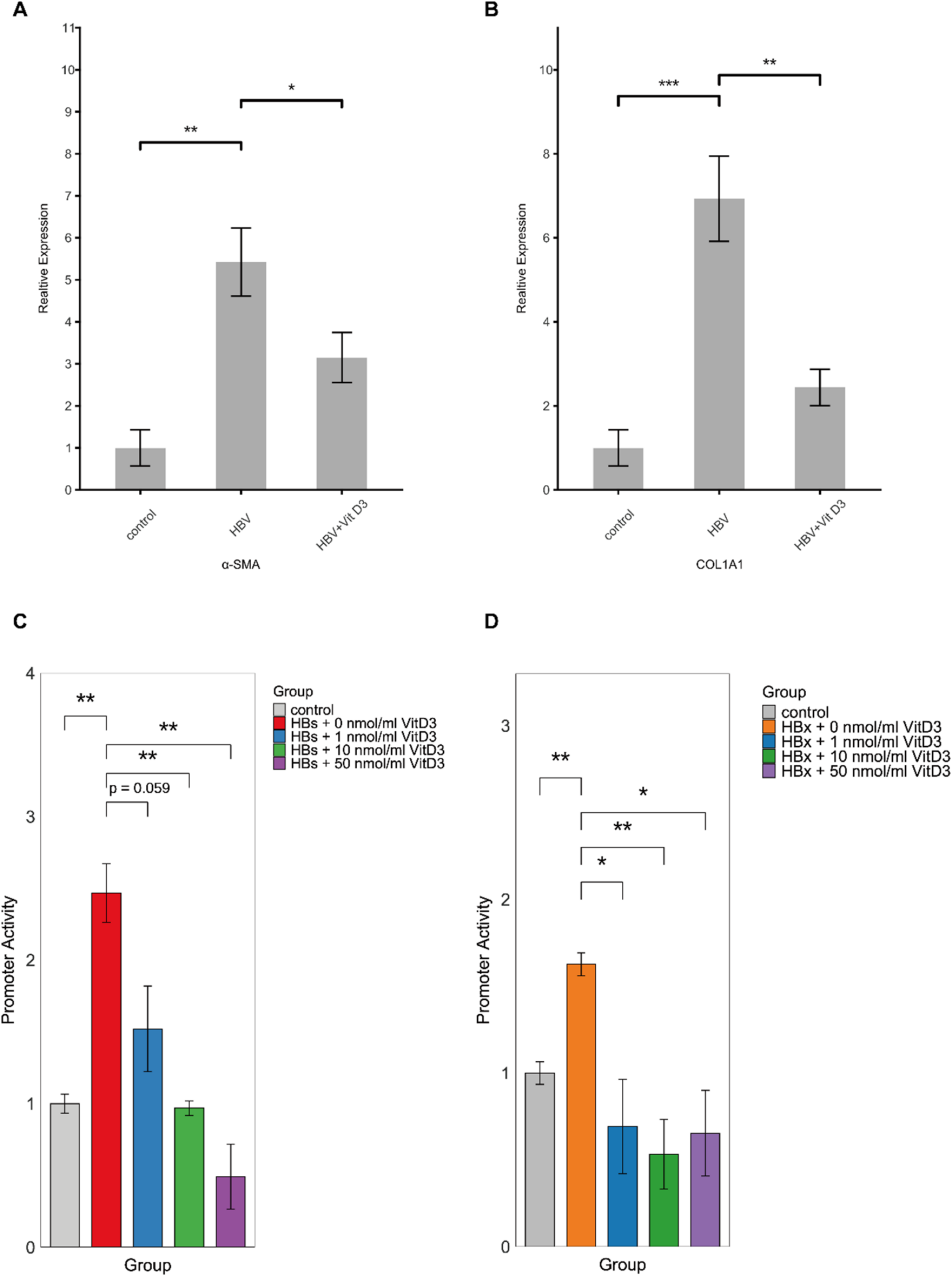
HBV activates HSCs through the RUNX2/ITGBL1 axis. Huh7 cells. (2 × 10⁵ cells/well) were cultured for 12 h and transfected with 2 µg of the 1.2 HBV expression plasmid. Vitamin D₃ (50 ng/mL) was also added. To collect conditioned medium for LX-2 culture, Huh7 and HepG2 cells were cultured in FBS-free Williams E medium for 48 h after plasmid transfection. LX-2 cells (2 × 10⁵ cells/well) were then cultured in hepatocyte cell supernatant for 48 h before harvesting. (**A**-**B**) CoL1A1 and α-SMA mRNA expression increased in the HBV expression medium and was downregulated after the addition of Vit D₃. (**C**-**D**) The ITGBL1 promoter plasmid and HBs and HBx expression plasmids were co-transfected into HEK293T cells (96-well plate), and increasing concentrations of Vitamin D3 were added. The transcriptional activity of the ITGBL1 promoter was detected by dual-luciferase reporter assays 48 h after co-transfection. The activity of the ITGBL1 promoter decreased as the concentration of Vitamin D₃ increased. The figure shows a representative image

In our previous study, we demonstrated that conditioned medium from ITGBL1-overexpressing cells could activate the human stellate cell line LX-2 [9]. To investigate whether HBV similarly promotes hepatic stellate cell (HSC) activation, we treated LX-2 cells with conditioned medium from HBV-transfected Huh7 cells and administered Vitamin D3 to Huh7 cells to assess its inhibitory effects. RT-qPCR analysis revealed that conditioned medium from HBV-transfected Huh7 cells significantly activated LX-2 cells, as evidenced by the upregulation of α-SMA and COL1A1 mRNA levels. Notably, treatment with Vitamin D3 effectively blocked this activation, suggesting that Vitamin D3 can inhibit HBV-induced ITGBL1 expression and subsequent HSC activation (Fig. 4A–B).

To further elucidate the mechanism by which HBV proteins regulate ITGBL1 expression, we employed dual-luciferase reporter assays. These results showed that both HBx protein and HBsAg increased the activity of the ITGBL1 promoter. However, this upregulation was significantly suppressed by Vitamin D3 treatment, indicating that HBV-mediated transcriptional activation of ITGBL1 is dependent on RUNX2 activity (Fig. 4C–D).

Overall, these results suggest that HBV promotes liver fibrosis by upregulating ITGBL1 expression through RUNX2, with viral proteins HBx and HBsAg playing crucial roles in this regulatory mechanism.

## Discussion

ITGBL1 promotes organ fibroblast behavior and the progression of tumorigenesis. In the current study, we describe the mechanism by which HBV induces ITGBL1 expression by elevating the transcription factor RUNX2, which in turn enhances ITGBL1 expression through direct binding to the ITGBL1 promoter.

Our data showed that HBV expression boosted ITGBL1 expression in vitro and in vivo. Up to now, there was no report on regulation of ITGBL1 in liver tissue. To explore this issue, we performed co-expression analysis using the expression profiling array of liver biopsy samples from CHB patients (dataset GSE84044) from our previous study, which included 124 CHB patients with different liver fibrosis stages. The analysis revealed significant co-expression of ITGBL1 with RUNX2. Then, ChIP assays combined with dual-luciferase reporter assays demonstrated that RUNX2 binds to the ITGBL1 promoter and boosts its activity. Knockdown of RUNX2 using shRNA significantly suppressed ITGBL1 expression by reducing ITGBL1 promoter activity. In contrast, overexpression of RUNX2 led to a significant increase in ITGBL1 protein levels. Moreover, the regulatory mechanism between RUNX2 and ITGBL1 has been previously reported in other contexts. For example, in breast cancer, RUNX2 was identified as a key transcription factor that regulates ITGBL1 expression, promoting the formation of osteomimetic breast cancers and metastasis to bone [15]. Similarly, a study on colorectal cancer (CRC) demonstrated that ITGBL1 was co-expressed with RUNX2 and that RUNX2 regulates the ITGBL1 promoter activity, further supporting the role of RUNX2 in regulating ITGBL1 expression [5]. Additionally, in melanoma, it was shown that RUNX2 silencing led to decreased ITGBL1 expression, emphasizing the importance of RUNX2 in regulating ITGBL1 in different cancer types [17]. A recent study also demonstrated that RUNX2 transcriptionally activates ITGBL1 in hepatocellular carcinoma, contributing to immune evasion in the tumor microenvironment [20]. These studies align with our findings, suggesting that RUNX2-mediated regulation of ITGBL1 might be a conserved mechanism across various diseases.

These results support our hypothesis that RUNX2 regulates ITGBL1 expression and that this pathway may be involved in the progression of liver fibrosis in chronic hepatitis B, as observed in our dataset and confirmed by previous research in other disease contexts. Furthermore, ITGBL1 expression was reduced in Huh7 cells treated with Vitamin D3, a RUNX2 inhibitor, consistent with decreased RUNX2 protein levels. Additionally, we provided evidence that HBV upregulates ITGBL1 expression at the transcriptional level in a RUNX2-dependent manner. RUNX2 expression was increased in HBV-Huh7 cells and HBV-replicative HepAD38 cells. The induction of ITGBL1 by HBV was blocked by Vitamin D3 and the RUNX2 inhibitor CADD522. Moreover, the elevation of ITGBL1 promoter activity due to HBV was blocked by RUNX2 knockdown. We also found that the viral proteins HBx and HBsAg increased ITGBL1 promoter activity, which could also be blocked by RUNX2 inhibitors, such as Vitamin D3.

These findings suggest that multiple HBV proteins are capable of modulating ITGBL1 transcriptional regulation. However, the underlying mechanisms remain to be fully elucidated. For HBsAg, the available evidence is insufficient to confirm a direct regulatory effect, and we speculate that its influence may be mediated through indirect mechanisms such as endoplasmic reticulum (ER) stress. Recent studies further support this view, showing that persistent ER stress driven by HBsAg accumulation contributes to hepatocarcinogenesis and reshapes transcriptional regulatory networks, which may also play a role in fibrogenic progression during HBV infection [21]. In contrast, HBx is a well-characterized viral regulator known to affect host gene expression through diverse pathways. Our ongoing investigations have identified a potential involvement of HBx in modulating upstream transcriptional regulators of ITGBL1, although the detailed mechanisms require further validation and will be reported in future studies. These findings are consistent with the notion that HBV-induced ITGBL1 upregulation may converge on transcriptional regulators such as RUNX2, underscoring the importance of the RUNX2/ITGBL1 axis in the progression of HBV-associated liver fibrosis.

To further investigate whether viral replication levels contribute to the activation of RUNX2 and ITGBL1 expression, we analyzed DNA levels in animal models. Notably, no significant correlation was observed between serum HBV DNA levels and hepatic ITGBL1 expression in the HBV mouse model, as assessed by both repeated measures correlation (RM-correlation) and linear mixed-effects model (LMM) analyses (Supplementary Figure S3A–D). Similarly, Pearson correlation analysis of liver tissue transcriptome data from CHB patients (GSE84044) revealed a statistically significant but weak positive correlation between HBV DNA levels and RUNX2 expression (*R* = 0.24, *p* = 0.01), while no significant correlation was observed for ITGBL1 expression (*R* = -0.11, *p* = 0.26) ), as shown in Supplementary Figure S3E–F. These findings suggest that HBV DNA levels alone may not fully reflect the molecular mechanisms driving RUNX2/ITGBL1 activation. This notion is further supported by a large clinical cohort study, which demonstrated a non-linear association between serum HBV DNA levels and liver fibrosis scores (e.g., APRI and FIB-4), with moderate viral loads showing the highest fibrosis indices [22]. Instead, HBsAg and HBx are more likely to serve as direct effectors of fibrogenic transcriptional regulation. In this study, due to the lack of HBsAg quantification in the clinical cohort [9], we were unable to further assess the relationship between viral protein levels and gene expression. Nevertheless, these data highlight the importance of considering HBV protein-mediated effects in the pathogenesis of liver fibrosis, beyond the influence of viral load alone.

Our data demonstrate that in liver cells, RUNX2 regulates ITGBL1 expression, and RUNX2 inhibitors can suppress ITGBL1 expression and block its downstream activation of HSCs under HBV replication conditions. RUNX2 has been reported as an oncoprotein and plays a role in promoting organ fibrosis. Studies have shown that RUNX2 drives the progression and bone metastasis of breast cancer by forming the NuRD (MTA1)/CRL4B complex, which represses the transcription of genes critical for cell growth, EMT, and invasion [23]. Conditional deletion of Runx2 markedly reduced extracellular matrix deposition and attenuated fibrosis progression [24]. These findings collectively highlight the pivotal role of RUNX2 in organ fibrogenesis beyond cancer biology. Recent studies have shown that RUNX2 can activate the TGF-β signaling pathway by upregulating Itgav expression in CCl4 and non-alcoholic steatohepatitis (NASH) mouse models [14]. Another study demonstrated that RUNX2 promotes liver fibrosis and HSC activation by increasing unconjugated cholic acid through binding the SLC27A5 promoter and downregulating SLC27A5 [13]. Our results similarly demonstrate that RUNX2 promotes liver fibrosis in the context of HBV expression. These findings are consistent with previous studies, indicating that RUNX2 promotes liver fibrosis by upregulating ITGBL1 expression.

Our data show that Vitamin D3, a RUNX2 inhibitor, attenuates HBV-induced ITGBL1 upregulation. Vitamin D3 deficiency is prevalent in CHB patients, with a 2013 study reporting that 81% of 203 patients had serum levels < 20 ng/ml. A negative correlation between Vitamin D3 levels and HBV DNA load has been consistently observed [25]. A multicenter study found 93% of 737 CHB patients had insufficient or deficient Vitamin D3 levels. Low Vitamin D levels correlate with worse prognoses [26]. A Hong Kong study following 426 patients for 13 years identified low Vitamin D3 as an independent predictor of adverse outcomes, including HCC and liver cirrhosis [27]. Our study results may partially explain the association between Vitamin D3 deficiency and poor prognosis. Additionally, we found that the viral proteins HBsAg and HBx are involved in the regulation of ITGBL1 and that this regulation can be blocked by Vitamin D3. This suggests that ITGBL1 regulation by HBV cannot be efficiently inhibited by antiviral therapy alone, as antiviral therapy, especially nucleos(t)ide analogue (NA) treatment, does not suppress viral protein expression directly. Therefore, combining antiviral therapy with RUNX2 inhibitors or Vitamin D3 supplementation may be a novel strategy to block the progression of liver fibrosis in CHB patients. The mechanism by which HBV suppresses serum Vitamin D3 levels needs further exploration.

Collectively, our data demonstrate that HBV induces ITGBL1 expression to activate HSCs by enhancing ITGBL1 promoter activity through elevated RUNX2 protein levels. Importantly, the Vitamin D3/RUNX2/ITGBL1 axis is a potent mechanism for regulating HSC activation. Targeting RUNX2 with Vitamin D3 or CADD522 may represent promising therapeutic strategies for preventing liver fibrosis progression in CHB patients. Taken together, our findings demonstrate that RUNX2 is a crucial regulator in liver fibrosis by activating HSCs. Importantly, the PKA/RUNX2/Itgav pathway is a probable mechanism for regulating HSC activation. Targeting RUNX2 may represent a promising therapeutic strategy for liver fibrosis.

## Conclusion

In this study, we identified a novel regulatory pathway through which hepatitis B virus (HBV) induces liver fibrosis via the RUNX2-mediated upregulation of integrin beta-like 1 (ITGBL1), which can be blocked by Vitamin D3. These insights contribute to the growing understanding of the molecular mechanisms underlying HBV-related liver fibrosis and offer valuable targets for therapeutic intervention.

## Electronic supplementary material

Below is the link to the electronic supplementary material.

Supplementary Material 1: Target Sequences for shRNAs Against RUNX2

Supplementary Material 2: Primer Sequences for qPCR and ChIP Assays

Supplementary Material 3: Correlation analyses between HBV DNA levels and ITGBL1/RUNX2 expression

Supplementary Material 4

## Data Availability

The datasets used and/or analysed during the current study are available from the corresponding author on reasonable request. The GSE84044 dataset can be accessed via GEO at https://www.ncbi.nlm.nih.gov/geo/query/acc.cgi? acc=GSE84044.

## Abbreviations

α-SMA: Alpha-smooth muscle actin
ANOVA: Analysis of variance
CADD522: RUNX2 inhibitor
CHB: Chronic hepatitis B
ChIP: Chromatin immunoprecipitation
ChIP-qPCR: Chromatin immunoprecipitation followed by quantitative PCR
COL1A1: type I collagen alpha 1 chain
CRL4B: Cullin 4B-RING E3 ligase complex
DMEM: Dulbecco’s Modified Eagle Medium
EGF: Epidermal growth factor
ELISA: Enzyme-linked immunosorbent assay
FBS: Fetal bovine serum
GAPDH: Glyceraldehyde 3-phosphate dehydrogenase
GEO: Gene Expression Omnibus
HBV: Hepatitis B virus
HBeAg: Hepatitis B e antigen
HBsAg: Hepatitis B surface antigen
HBx: Hepatitis B virus X protein
HCC: Hepatocellular carcinoma
HSC: Hepatic stellate cell
IHC: Immunohistochemistry
ITGBL1: Integrin beta-like 1
LMM: Linear mixed-effects model
LX-2: Human hepatic stellate cell line
MOCK: Mock-infected control
NAFLD: Nonalcoholic fatty liver disease
NASH: Nonalcoholic steatohepatitis
NuRD: Nucleosome remodeling and deacetylase
ORF: Open reading frame
qPCR/RT-qPCR: (reverse transcription) quantitative polymerase chain reaction
R: R Programming language
RM-correlation: Repeated measures correlation
rAAV: Recombinant adeno-associated virus
RUNX2: Runt-related transcription factor 2
SD: Standard deviation
SEM: Standard error of the mean
shRNA: Short hairpin RNA
TGF-β: Transforming growth factor-beta
VitD₃: 25-dihydroxyvitamin D3
Vge: Viral genome equivalents
