# Supplementary material for: HBV activates hepatic stellate cells through RUNX2/ITGBL1 axis
Source: Virol J. 2025 Apr 26;22:120. doi: 10.1186/s12985-025-02749-z (PMC12032756; doi:10.1186/s12985-025-02749-z)
Supplement: Supplementary file 3 — Supplementary Material 3: Correlation analyses between HBV DNA levels and ITGBL1/RUNX2 expression [file 12985_2025_2749_MOESM3_ESM.pdf]

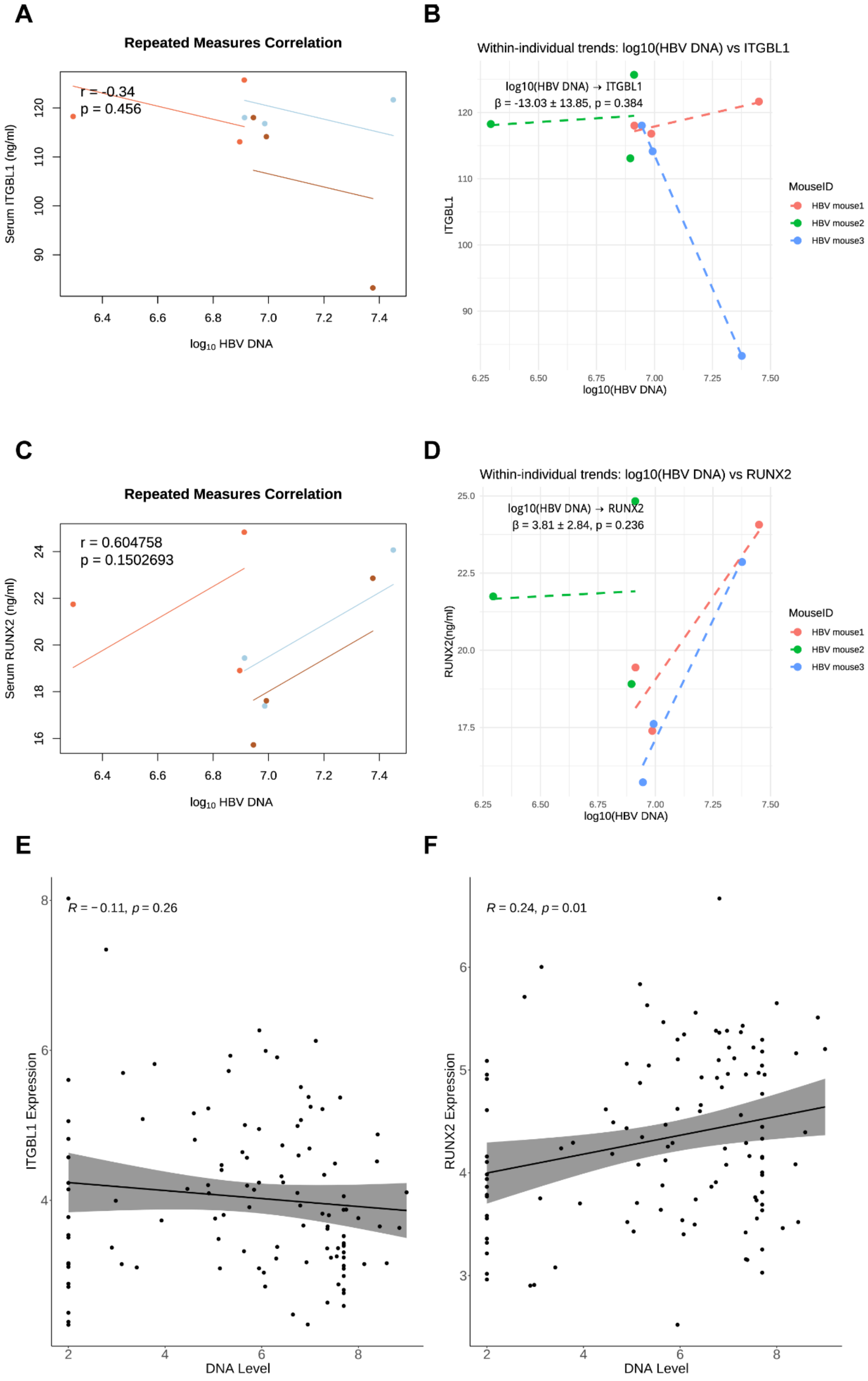

Supplementary Figure 3. Correlation analyses between HBV DNA levels and ITGBL1/RUNX2 expression

(A–B) Correlation between serum HBV DNA levels and ITGBL1 expression in HBV-infected mice, assessed using repeated measures correlation (A) and linear mixed-effects model (LMM) (B).

(C–D) Correlation between serum HBV DNA levels and RUNX2 expression in HBV-infected mice, analyzed by repeated measures correlation (C) and LMM (D).

(E–F) Pearson correlation analysis using liver tissue transcriptome data from HBV-infected patients (GSE84044), showing the correlation between HBV DNA levels and ITGBL1 (E) or RUNX2 (F) expression.
