# Supplementary figures and images for: HBV activates hepatic stellate cells through RUNX2/ITGBL1 axis
Source: Virol J. 2025 Apr 26;22:120. doi: 10.1186/s12985-025-02749-z (PMC12032756; doi:10.1186/s12985-025-02749-z)

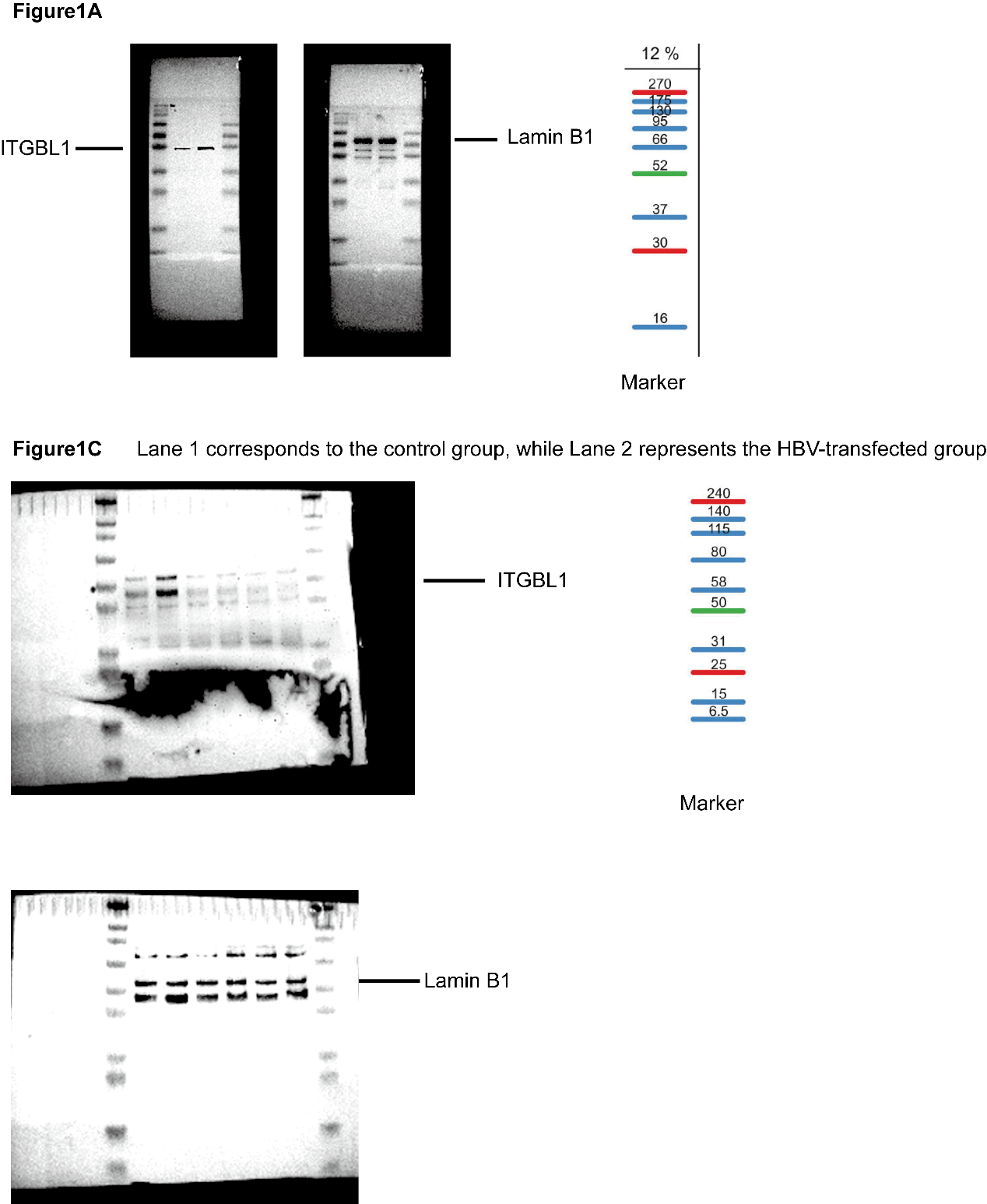


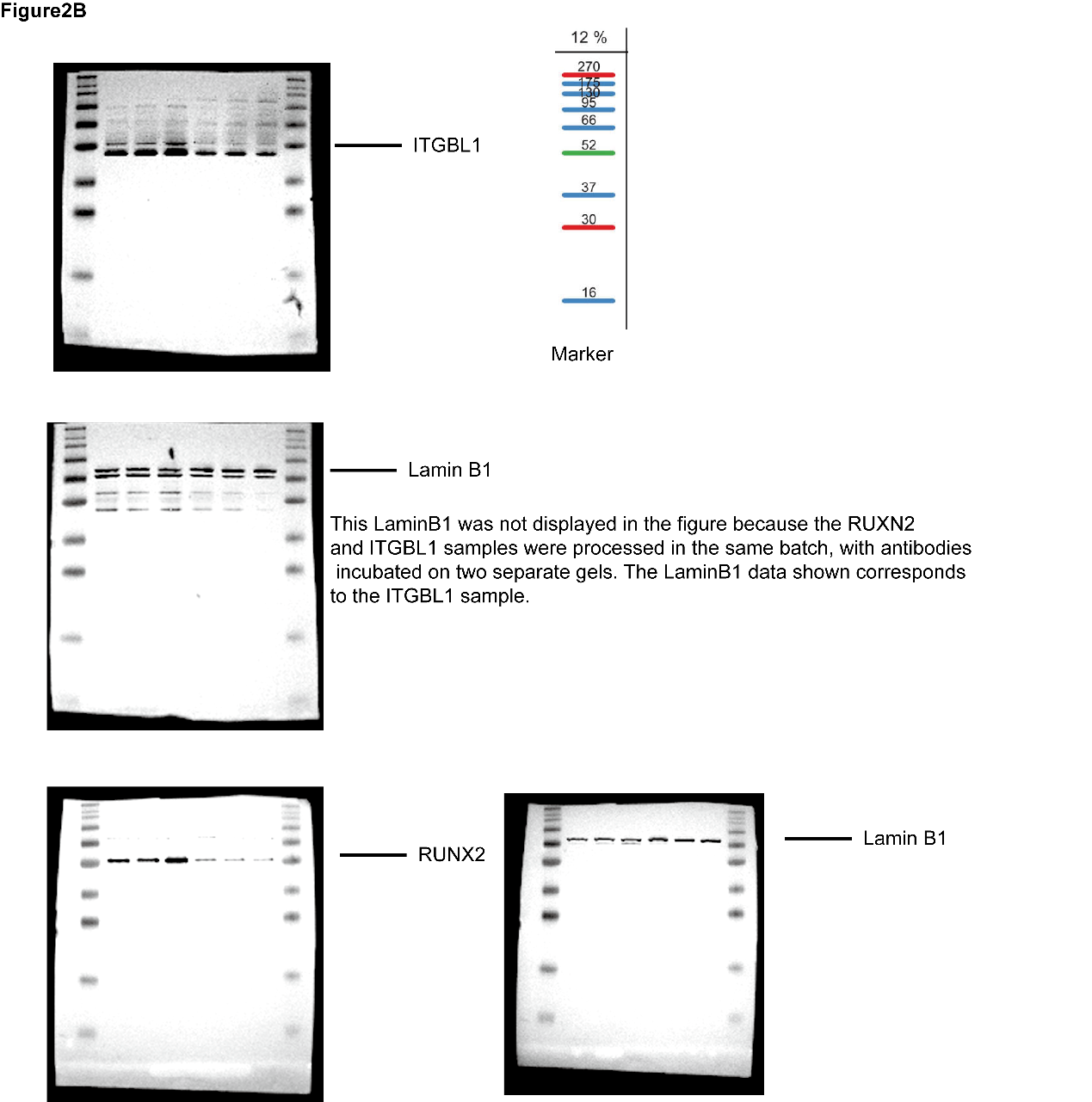


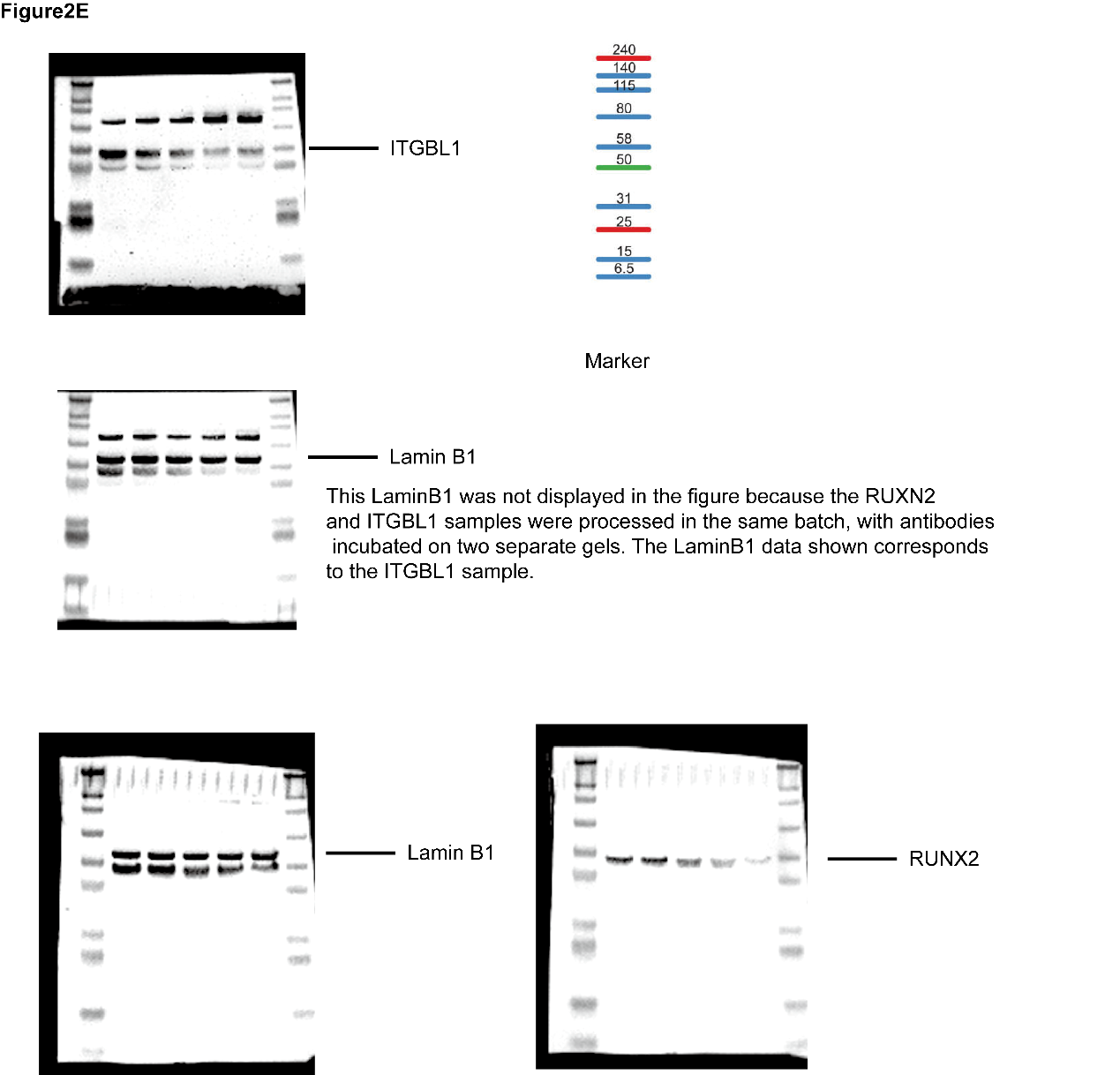


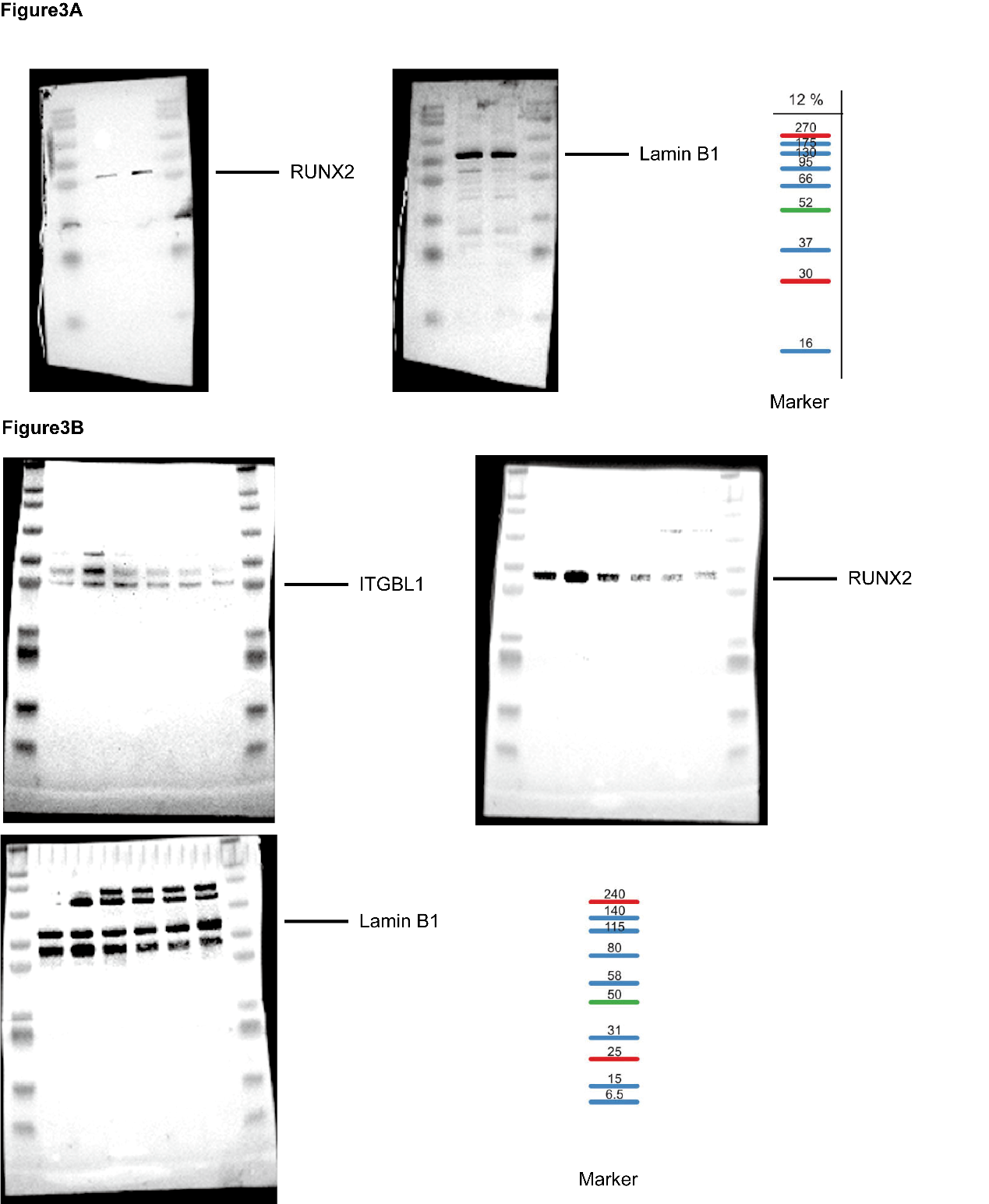


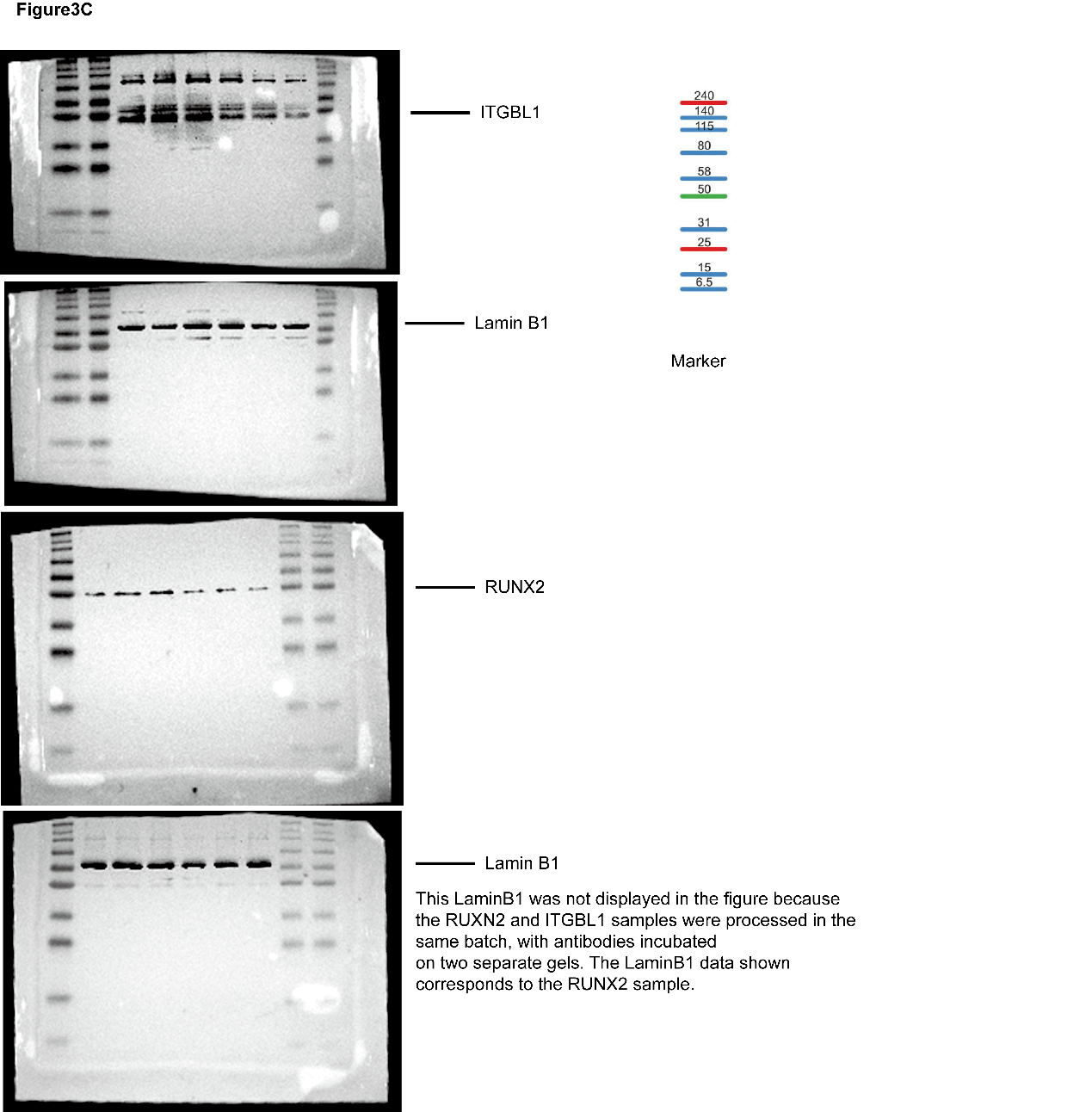

Supplement: Supplementary file 4 — Supplementary Material 4 [file 12985_2025_2749_MOESM4_ESM.docx]
